# An optimal growth pattern during pregnancy and early childhood associates with better fertility in men

**DOI:** 10.1530/EJE-22-0385

**Published:** 2022-10-13

**Authors:** Johanna Laru, Marja Ojaniemi, Stephen Franks, Marjo-Riitta Järvelin, Elisa Korhonen, Terhi T Piltonen, Sylvain Sebert, Juha S Tapanainen, Laure Morin-Papunen

**Affiliations:** 1 Department of Obstetrics and Gynecology, University of Oulu and Oulu University Hospital, Medical Research Center, PEDEGO Research Unit, Oulu, Finland; 2 Department of Children and Adolescents, University of Oulu and Oulu University Hospital, Medical Research Center, PEDEGO Research Unit, Oulu, Finland; 3 Institute of Reproductive and Developmental Biology, Imperial College London, London, UK; 4 Center for Life Course Health Research, University of Oulu, Oulu, Finland; 5 Department of Life Sciences, College of Health and Life Sciences, Brunel University, London, UK; 6 Unit of Primary Health Care, Oulu University Hospital, Oulu, Finland; 7 Department of Epidemiology and Biostatistics, MRC-PHE Centre for Environment and Health, School of Public Health, Imperial College London, London, UK; 8 Department of Obstetrics and Gynecology, University of Helsinki and Helsinki University Hospital, Helsinki, Finland

## Abstract

**Objective:**

This study aimed to evaluate the association between birth weight (BW), childhood and adolescent BMI, with reproductive capacity in men.

**Design:**

A prospective, population-based cohort study (Northern Finland birth cohort 1966).

**Methods:**

Around 6196 men born in 1966 were followed from birth to age 50 years. Weight and height were measured repeatedly by professionals. Reproductive capacity (infertility assessment, male factor infertility and infertility treatment by age 46 years) was evaluated by questionnaires at ages 31 and 46 years. The number of children by the age of 50 years was recovered from registers. After excluding the men who reported never having attempted to have children or not answering the question at age 31 or 46 years (*n* = 2041), 4128 men were included in the final study population. Results were adjusted for BW, BW for gestational age (GA), mother’s smoking status, marital status, educational level and smoking status.

**Results:**

Being small for GA (10.5% vs 8.2%, *P* = 0.012) or having a lower BW (3495 g vs 3548 g, *P* = 0.003) were associated with childlessness. The association was however no longer significant after adjusting for marital status. Being underweight in early childhood was associated with an increased risk of infertility assessment (adjusted, aOR: 2.04(1.07–3.81)) and childlessness (aOR: 1.47(1.01–2.17)) compared to the normal weight group. Conversely, overweight or obesity in early childhood was associated with a decreased risk of infertility assessment (aOR: 0.60 (0.41–0.87)), treatment (aOR: 0.42 (0.25–0.70)) and male factor infertility (aOR: 0.45 (0.21–0.97)). BMI in mid-childhood or puberty had no association with infertility or childlessness.

**Conclusion:**

In boys, an optimal growth trajectory during pregnancy and early childhood seems to be very important for life-long fertility.

## Introduction

Obesity is a worldwide pandemic, and the increasing rate of childhood obesity is especially concerning as it increases the risk of long-term health problems (1, 2). Adult obesity is a risk factor for infertility and childlessness not only in women (3, 4) but also in men (5, 6). Infertility affects approximately 15% of the couples trying to conceive and male infertility accounts for 20–30% of all infertility causes and, in combination with female factors, for another 30% (7, 8). There are three main causes of male infertility: obstruction of seminal outflow, hypothalamic–pituitary disease (secondary hypogonadism) and testicular dysfunction (primary hypogonadism) (8, 9). Obesity is linked to the latter two conditions (5, 6, 10), but an association between low BMI and fertility is under debate (11, 12).

To our knowledge, only two previous studies have been conducted in adolescent boys, showing an inverted J-shaped relationship between BMI and later reproductive capacity (13, 14). Studies concerning association between childhood growth and testicular function have shown that unfavorable fetal growth patterns, especially being small for gestational age (SGA) (15, 16, 17, 18) and non-optimal growth and high adiposity in childhood, are associated with impaired testicular function in adulthood (17). Typically, BMI increases from birth and reaches a maximum at age 7–9 months (adiposity peak, AP), after which it decreases, reaching its nadir at age 4–6 years (adiposity rebound, AR) (19, 20, 21). Early AR, occurring before 5 years (21), has been correlated with obesity and a poor metabolic profile in adulthood (20, 21).

This study is an extension of our previous study on women’s childhood growth and fertility (22). The aim of the study was to evaluate, in men, the association between childhood underweight, overweight and obesity with adulthood fertility among three age groups (early childhood, mid-childhood and puberty) and the association of BMI and timing of AP and AR with infertility parameters. Reproductive capacity was evaluated by assessing the occurrence of investigation and treatment for infertility, and male factor infertility by age 46 years, as well as childlessness and number of children by age 50 years.

## Methods

**Figure 1:**
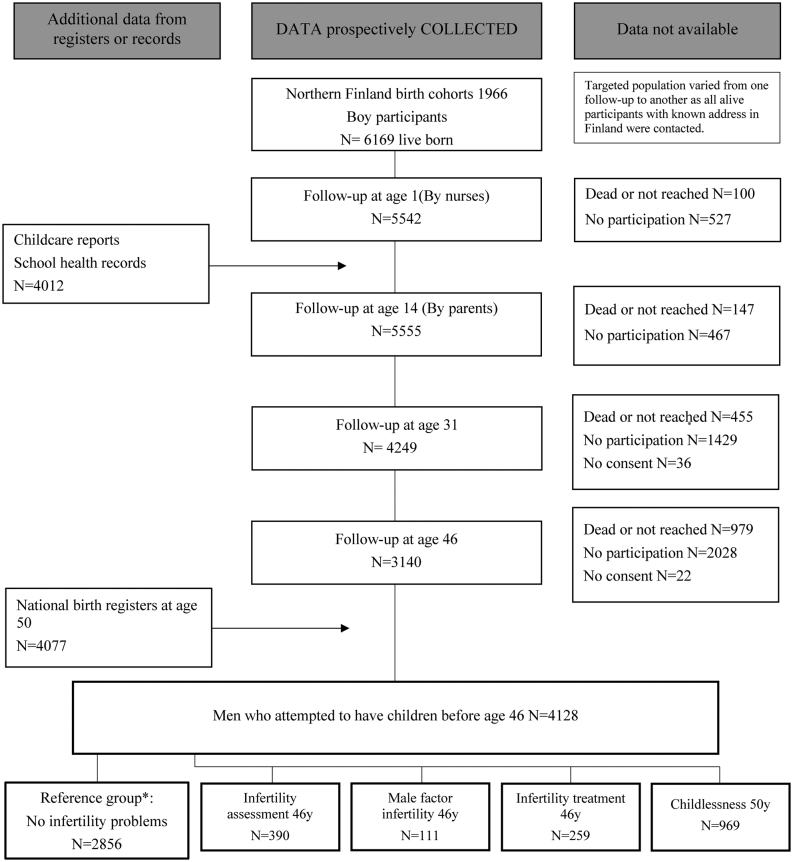
Flowchart of the study population.

The study population was drawn from a general population birth cohort, the Northern Finland Birth Cohort 1966 (NFBC1966), recruited at gestational week 24 from the two northernmost provinces of Finland. The study included a total of 12 058 live births (6169 males), covering 96% of all births in this area (23). The study was approved by the Ethics Committee of the Northern Ostrobothnia Hospital District. All participants provided informed consent. (Fig. 1)

The questionnaires for ages 31 and 46 years included questions on lifestyle, education, family history, health and fertility (Supplementary Table 1, see section on supplementary materials given at the end of this article). To the following question: ‘Have you ever attempted to have children?’ 4026 men answered at age 31 years and 3012 at age 46 years. Of these, 3372 and 2802 had attempted to have children by 31 or 46 years, respectively, and the final study population included 4128 men who had attempted to have children by age 46 years (Fig. 1, Supplementary Table 1). In total, 322 men reported that they had never attempted to have children and they were excluded from the study.

The outcomes concerning infertility were defined based on the answers to the questionnaires at 31 and 46 years (Supplementary Table 1). The number of childbirths recorded up to the end of 2016 (when the men turned 50 years) was obtained from the Finnish Medical Birth Register, which was established in 1987. Birth data before 1987 were collected from the Population Register Center live births to make the birth data complete. The final study population was divided into five groups: infertility assessment (*n* = 390), male factor infertility (*n* = 111), infertility treatment (*n* = 259), childlessness (*n* = 969) and the reference group which comprised men without any fertility problem (*n* = 2856) (Fig. 1). Men without any fertility problems were used as a reference group in all analyses.

### Growth data and definition of obesity and underweight in the study population

The weight and height of the children aged 6 and 12 months were measured by experienced nurses. The BMI of children aged 6 months (*n*  = 2725) and 12 months (*n*  = 3513) was analyzed separately. Data on weight and height from infancy to adolescence were collected from measurements reported by the child health and welfare nurses, and later by the school nurses, as part of the national child health program, which is free for all children in Finland.

Three different age groups were created: early childhood (from infancy to adrenarche: 3.00–6.99 years, *n*  = 2603), mid-childhood (juvenile stage: 7.00–10.99 years, *n*  = 2826) and puberty (adolescence: 11.00–15.99 years, *n*  = 2927), according to the literature regarding childhood growth (24, 25) and physiological hormonal changes (adrenal cortex activation and gonadal maturation) occurring during childhood until adolescence (26, 27). If participants had more than one measurement per year, the mean BMI for that year was calculated. Median number of measurements per age period (range) was two in early childhood (0–12), two in mid-childhood (0–12) and three in puberty (0–18). For each age group, the participants were stratified into underweight (below 5th percentile (pc)), normal weight (5th–85th pc), overweight (85th–95th pc) and obese (over 95th pc) categories according to the criteria of the World Health Organization (WHO) and the Center for Disease Control and Prevention (CDC) (28). Being obese or underweight in any of the specified age groups was defined by the occurrence of at least one annual BMI value over the 95th pc or under the 5th pc, respectively. This categorization has been widely used in studies concerning fertility, hormonal parameters and childhood growth (13, 14, 17, 22, 29, 30).

The timing of AP and AR was derived from fitted growth curves, as described previously, and for each participant, predicted BMI at AP and AR was calculated using the estimated fixed and random coefficients (31). The normal changes observed in childhood BMI required the data to be split into two age windows: infancy (2 weeks to 1.5 years) and childhood (1.5 to 13 years). There were, on average, 7 measurements during infancy and 16 during childhood for each child. Calculation of AP (*n* = 2339) and AR (*n* = 2922) was made only for the children having at least three measurements during childhood.

Height and weight at age 14 were all measured and reported by the parents (*n* = 3644) and used only for continuous analyses. There was no statistical difference between measured (*n* = 2401) and self-reported BMI at age 14 years (mean BMI, 19.35 vs 19.31 kg/m^2^). Height and weight at age 31 years were measured at the clinical examination. If measurements were not available, self-reported data were used. There was no statistically significant difference between self-reported (25.51 kg/m^2^) and clinically measured (25.30 kg/m^2^, *P* = 0.103) BMI.

### Covariates

Prenatal factors (maternal age, smoking at the end of pregnancy, pre-pregnancy BMI), birth weight (BW), birth length (BL), BW−BL ratio, BW for gestational age (GA) and GA were included in the background factors as they are known to be associated with childhood growth (32) and childlessness (15, 33). Data on BW, BL and GA (defined from the last menstrual period) were collected by local midwives in the antenatal clinics. Individuals with BW under the 10th pc for GA were considered as small for GA (SGA) and individuals with BW over the 90th pc for GA were considered as large for GA (LGA). Marital status was defined as either ‘being single’ (both at ages 31 and 46 years) or ‘ever being in long relationship’ (ever married/cohabited/divorced/widowed). Alcohol consumption, smoking and educational level (used as a proxy for socioeconomic status in this cohort) at age 31 years were classified based on the questionnaire (Table I online only). According to a directed acyclic graph (Supplementary Fig. 1), BW, BW for GA, mother’s smoking and marital status, as well as smoking and educational level at age 31 years, were selected for the adjustment models (www.dagitty.net).

### Statistical methods

Differences in the continuous variables were analyzed by an independent *t*-test, a Mann–Whitney *U* test, a one-way ANOVA or a Kruskal–Wallis test, as appropriate. To assess differences in the categorical parameters, a chi-square test was used. For these tests, results were reported as means or medians, standard deviations (s.d.) or prevalence (%) and odds ratios (OR) with a 95% CI, respectively. A *P*-value <0.05 was considered statistically significant. Bonferroni correction was used for additional analyses in different age groups. Multivariable analyses were conducted using binary logistic regression modeling. The results were reported as OR with 95% CI. Linear and quadratic associations between the number of children (the birth of the sixth or any subsequent child was not considered since only 2.9% of the participants had more than six children) and BW and BMI at 14 years were assessed with regression models. IBM SPSS Statistics for Windows, Version 25.0. (Armonk, NY: IBM Corp.) was used to assess differences between the study groups and to perform the regression analyses.

## Results

### Characteristics of the study population

The number of participants varied between each age group and different fertility outcomes, owing to variations in the number of answers to each question and/or participation in the clinical examination (Table 1). In all, 1272 (30.8%) men reported a fertility problem (infertility assessment or treatment) or remained childless (Fig. 1). Men who had children were more likely to have ever been in a relationship compared with their counterparts. Men who underwent infertility assessment and treatment were more often non-smokers and had higher education level. Men with male factor infertility had higher education level, but their smoking habits did not differ. In contrast, childless men had lower education level, but they also smoked less. Alcohol consumption was not associated with any of the infertility variables (Table 1).

**Table 1:** Characteristics of the study population. Men who reported never having attempted to have children were excluded from the analyses. ‘No infertility problems’ was used as a reference group. Data are presented as mean ± s.d. or as percentages.

	No infertility problems^a^	Infertility assessment before age 46 years	Male factor infertility before age 46 years	Infertility treatments before age 46 years	Childlessness by age 50 years
*n*	2856	390	111	259	969
Mother’s parameters					
Mother’s prepregnancy BMI (kg/m^2^)	23.87 ± 9.84	23.39 ± 9.61	22.72 ± 10.75	23.99 ± 9.61	23.63 ± 9.15
Mother’s age (years)	27.99 ± 7.12	28.34 ± 7.22	26.91 ± 6.86	28.08 ± 6.85	28.54 ± 6.94
Mother’s smoking at the end of pregnancy (%)	14.5	14.1	19.6	17.8	16.8
Index person’s parameters					
Childhood					
Birth weight (g)	3548 ± 536	3555 ± 540	3524 ± 559	3560 ± 564	3495 ± 559^*^
Birth length (cm)	50.25 ± 5.52	50.83 ± 3.33	50.90 ± 2.26	50.93 ±2.13	50.18 ± 4.85
Birth weight–length ratio (g/cm)	70.34 ± 8.36	70.18 ± 8.78	69.66 ± 8.66	79.34 ± 8.98	69.46 ± 8.73^*^
Size for gestational age (%)					
SGA	8.2	9.1	10.0	8.2	10.5^*^
AGA	80.4	78.9	80.0	79.7	80.0
LGA	11.4	12.0	10.0	12.1	9.5
Gestational age (week)	40.01 ± 1.88	40.07 ± 1.81	39.91± 1.85	40.09 ± 1.80	39.89 ± 1.92
Prematurity (born before 37th GW) (%)	4.4	3.9	6.4	3.9	5.8
BMI 6 months (kg/m^2^)	17.93 ± 1.60	17.79 ± 1.50	17.87 ± 1.56	17.74 ± 1.54	17.80 ± 1.69
BMI 1 year (kg/m^2^)	18.07 ± 1.61	18.05 ± 1.56	18.11 ± 1.46	17.97 ± 1.58	17.99 ± 1.56
Age AP (months)	9.09 ± 0.43	9.10 ± 0.37	9.11 ± 0.39	9.09 ± 0.38	9.07 ± 0.35
BMI AP (kg/m^2^)	18.23 ± 0.79	18.15 ± 0.75	18.16 ± 0.74	18.07 ± 0.75*	18.11 ± 0.77^*^
Age AR (years)	5.72 ± 0.81	5.68 ± 0.78	5.70 ± 0.80	5.73 ± 0.81	5.67 ± 0.92^*^
BMI AR (kg/m^2^)	15.47 ± 0.94	15.48 ± 0.94	15.35 ± 0.86	15.37 ± 0.90	15.43 ± 1.00
BMI 14 years (kg/m^2^)	19.28 ± 2.47	19.31 ± 2.24	19.21 ± 2.58	19.19 ± 2.09	19.43 ± 2.81
Adulthood					
Ever been in long relationship (%)	94.6	97.9^**^	96.7	97.7^**^	50.1^**^
BMI 31 years (kg/m^2^)	25.38 ± 3.49	25.12 ± 3.15	25.45 ± 3.69	25.13 ± 3.04	25.42 ± 4.05
BMI 46 years (kg/m^2^)	27.32 ± 4.15	27.03 ± 3.29	27.87 ± 5.22	27.17 ± 4.14	27.63 ± 4.74
Alcohol consumption 31 years (g/day)	14.35 ± 21.90	11.85 ± 15.50	13.53 ± 21.45	12.24 ± 17.15	16.88 ± 29.76
Smoking 31 years (%)					
Non-smoker	35.0	44.2^*^	36.3	44.3^*^	40.8^*^
Former/occasional smoker	28.6	27.8	25.5	30.3	22.6^*^
Active smoker	36.4	28.0^*^	38.2	25.4^*^	36.6
Education 31 years (%)					
Basic	12.2	6.4^*^	9.6*	7.3^*^	14.2
Secondary	71.8	71.1	71.3	68.2	73.1
Tertiary	15.9	22.4^*^	19.1^*^	24.5^*^	12.7^*^
Number of children	2.65 ± 1.67	1.61 ± 1.53^**^	1.57 ± 1.60^**^	1.58 ± 1.53^**^	NA

^*^P < 0.05; ^**^P < .001 compared with the reference group;

^a^No infertility problems is defined as men without childlessness, infertility assessment and treatment

AGA, appropriate for gestational age; GW, gestational week; LGA, large for gestational age; NA, not applicable; SGA, small for gestational age.

### Prenatal factors and birth weight

**Figure 2:**
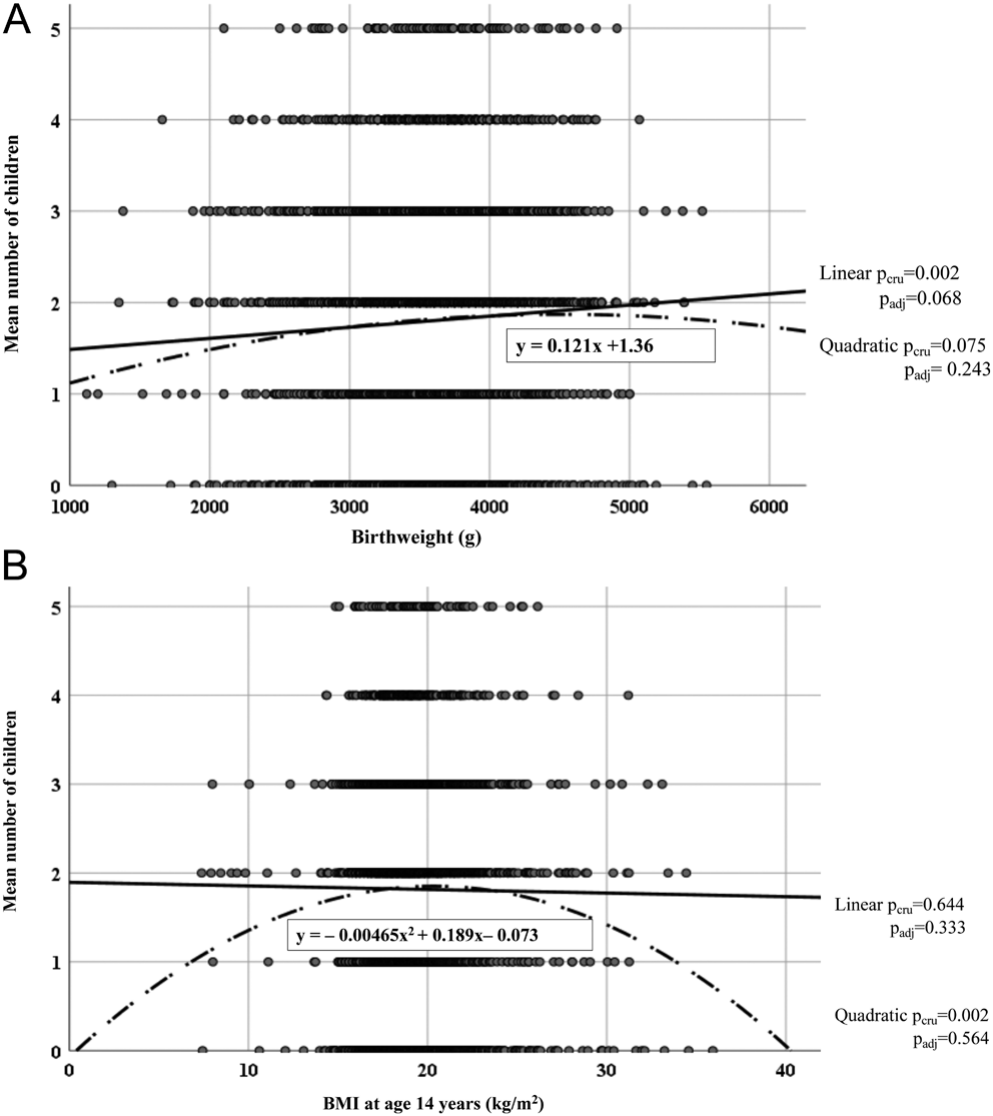
(A and B) Curve estimation between birth weight (BW) (A) and BMI at age 14 years (B), and number of children by age 50. Men who reported never having attempted to have children were excluded. Both models were adjusted for mother’s smoking, marital status until age 46 and education and smoking at age 31. Model B was also adjusted for BW and BW for gestational age.

Prenatal factors did not associate with any of the infertility variables or childlessness (Table 1). No associations between BW or SGA and infertility assessment, treatment or male factor infertility were found. SGA was associated with an increased risk of childlessness when compared to the appropriate for GA (OR_SGA_: 1.39 (1.06–1.83), *P* = .019) (Table 1). BW and BW−BL ratio were inversely associated with childlessness (Table 1) and BW with number of children in adulthood (Fig. 2A). After adjusting with marital status, however, the associations between BW and childlessness (adjusted aOR_BW_: 0.98 (0.97–1.01), *P* =.124), between BW−BL ratio and childlessness (aOR_BW–BL ratio_: 0.99 (0.98–1.01), *P* = 0.345) and between SGA and childlessness (aOR_SGA_: 1.23 (0.88–1.72), *P* = .210) lost their significance. A linear trend for a positive association between BW and number of children remained, even after adjusting for marital status in the regression analysis (Fig. 2A).

### Infancy

BMI at ages 6 and 12 months did not associate with infertility assessment, treatment, male factor infertility or childlessness by age 50 (Supplementary Table 2).

### AP and AR

Lower BMI at AP, but not age, was associated with childlessness, but the significance was lost after adjusting for marital status. BMI at AR did not associate with infertility variables or childlessness. Age at AR did not associate with infertility variables, but AR at an earlier age associated with an increased risk of childlessness after adjustments (Table 2).

**Table 2:** Association between age and BMI at adiposity peak/rebound and fertility outcomes. Men who reported never having attempted to have children were excluded from the analyses. ‘No infertility problems’ were used as a reference group. Results are shown as odds ratios (OR) with 95% CI.

	Infertility assessments before age 46	Male factor infertility before age 46 years	Infertility treatments before age 46	Childlessness by age 50
Adiposity peak, *n*	1872	1700	1790	2157
Age				
Crude	0.93 (0.64–1.36)	0.94 (0.45–1.95)	0.80 (0.49–1.30)	0.84 (0.64–1.11)
Model I	0.91 (0.62–1.34)	0.93 (0.43–1.94)	0.80 (0.49–1.31)	0.84 (0.63–1.11)
Model II	0.91 (0.63–1.34)	0.93 (0.44–1.93)	0.79 (0.49–1.29)	0.93 (0.66–1.29)
Model III	0.90 (0.60–1.31)	0.90 (0.45–1.92)	0.77 (0.47–1.26)	0.92 (0.65–1.28)
BMI				
Crude	0.94 (0.78–1.13)	0.97 (0.68–1.38)	0.82 (0.65–1.03)	0.83 (0.72–0.96)^*^
Model I	0.94 (0.77–1.12)	0.99 (0.67–1.41)	0.82 (0.64–1.02)	0.82 (0.71–0.95)^*^
Model II	0.94 (0.78–1.12)	0.98 (0.68–1.41)	0.82 (0.65–1.03)	0.86 (0.72–1.01)
Model III	0.95 (0.79–1.15)	1.00 (0.69–1.44)	0.83 (0.66–1.04)	0.85 (0.72–1.01)
Adiposity rebound, *n*	2342	2128	2234	2698
Age				
Crude	0.89 (0.76–1.05)	0.87 (0.64–1.18)	0.93 (0.76–1.14)	0.92 (0.84–1.04)
Model I	0.89 (0.77–1.05)	0.84 (0.62–1.16)	0.93 (0.75–1.15)	0.91 (0.81–1-03)
Model II	0.90 (0.76–1.06)	0.85 (0.62–1.15)	0.94 (0.77–1.15)	0.83 (0.72–0.96)
Model III	0.87 (0.74–1.04)	0.84 (0.61–1.15)	0.91 (0.74–1.12)	0.85 (0.74–0.97)^*^
BMI				
Crude	0.96 (0.83–1.10)	0.85 (0.63–1.14)	0.91 (0.75–1.10)	0.95 (0.85–1.04)
Model I	0.95 (0.84–1.11)	0.86 (0.65–1.16)	0.91 (0.75–1.10)	0.96 (0.86–1.08)
Model II	0.95 (0.83–1.11)	0.86 (0.65–1.17)	0.91 (0.76–1.09)	1.01 (0.89–1.12)
Model III	0.96 (0.83–1.11)	0.88 (0.66–1.17)	0.92 (0.76–1.10)	1.01 (0.90–1.14)

^*^Statistical significance after Bonferroni correction.

Model I: adjustment for birth weight, birth weight for gestational age and mother’s smoking at the end of pregnancy;

Model II: Model I + adjustment for marital status until age 46;

Model III: Model II + adjustment for education and smoking at age 31.

### Early childhood

Only 5.4% of boys who were underweight in early childhood and 23% of boys who were obese in early childhood remained underweight or obese in adulthood, respectively.

**Figure 3:**
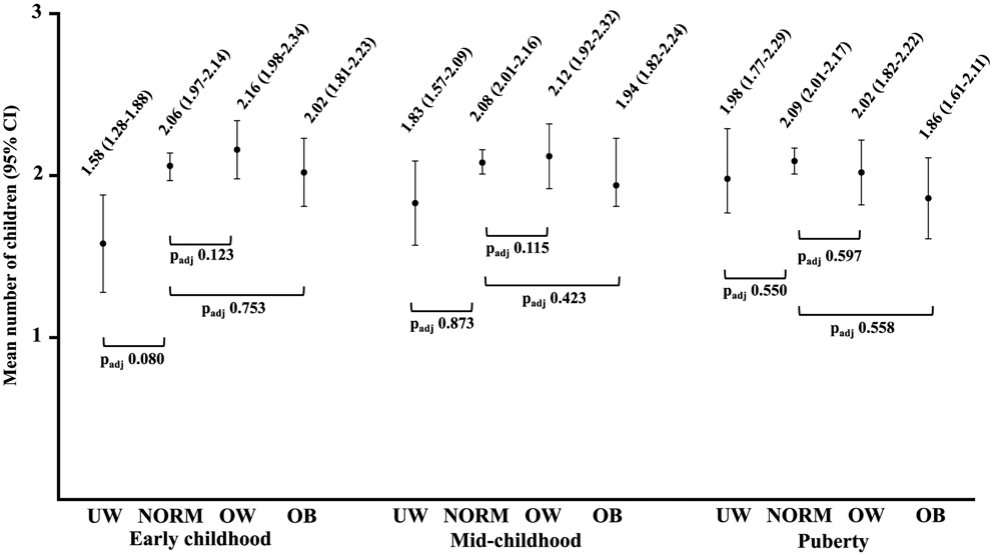
Total number of children by age 50 in early childhood, mid-childhood and puberty. Men who reported never having attempted to have children were excluded from the analyses. The results were adjusted for birth weight (BW), BW for gestational age, mother’s smoking, marital status until age 46 and education and smoking at age 31. Normal weight is used as a reference group. Adj, adjusted; y, year; UW, underweight (BMI < 5th percentile (pc)); NORM, normal weight (BMI 5th–85th pc); OW, overweight (BMI 85th–95th pc); OB, obese (BMI > 95th pc).

Being underweight in early childhood was associated significantly with childlessness (Table 3) and with a tendency for having fewer children by age 50 years, after adjustments (Fig. 3). In contrast, overweight was associated with a decreased risk of being assessed for infertility and being diagnosed for male factor infertility. Both overweight and obesity were associated with a lower risk of infertility treatment but not with childlessness or number of children (Table 3). When combining boys with overweight and obesity into a same group, a decreased risk of infertility assessment, male factor infertility and infertility treatment was noted (aOR: 0.60 (0.41–0.87), *P* = .002; 0.45 (0.21–0.97), *P* = .040 and 0.42 (0.25–0.70), *P* = .001, respectively). There was neither association with childlessness (aOR: 0.93 (0.69–1.25), *P* = .643) nor any significant difference in number of children when compared to normal weight boys (2.10 vs 2.09, *P* = .843).

**Table 3:** Association between childhood underweight, overweight and obesity in different age groups and the fertility outcomes compared to the normal weight boys. Men who reported never having attempted to have children were excluded from the analyses. ‘No infertility problems’ were used as a reference group. Results are shown as OR with 95% Cl.

	No infertility problems^a^	Infertility assessments before age 46	Male factor infertility before age 46 years	Infertility treatments before age 46	Childlessness at age 50
Early childhood, *n*	1838	260	70	160	560
Underweight					
*n* (%)	56 (3.0)	13 (5.0)	8 (11.4)	7 (4.4)	31 (5.5)
Crude	Ref	1.85 (0.99–3.45)	1.37 (0.61–3.10)	1.37 (0.79–2.39)	2.00 (1.45–2.72)^*^
Model I	Ref	1.45 (0.91–2.31)	1.37 (0.60–3.12)	1.44 (0.82–2.53)	1.92 (1.40–2.63)^*^
Model II	Ref	1.50 (0.94–2.39)	1.35 (0.59–3.09)	1.48 (0.84–2.59)	1.49 (1.02–2.19)
Model III	Ref	1.55 (0.97–2.49)	1.37 (0.60–3.15)	1.54 (0.87–2.73)	1.47 (1.01–2.17)
Overweight					
*n* (%)	302 (16.4)	32 (12.3)	6 (8.6)	15 (9.4)	79 (14.1)
Crude	Ref	0.69 (0.47–1.02)	0.37 (0.15–0.94)	0.42 (0.23–0.76)^*^	0.88 (0.67–1.15)
Model I	Ref	0.67 (0.45–0.99)	0.38 (0.15–0.96)	0.41 (0.23–0.73)^*^	0.89 (0.68–1.17)
Model II	Ref	0.67 (0.45–0.99)	0.38 (0.15–0.96)	0.41 (0.23–0.74)^*^	0.89 (0.65–1.23)
Model III	Ref	0.65 (0.44–0.97)	0.37 (0.14–0.93)	0.38 (0.21–0.69)^*^	0.88 (0.64–1.22)
Obese					
*n* (%)	199 (10.8)	19 (7.3)	5 (7.1)	10 (6.3)	62 (11.1)
Crude	Ref	0.56 (0.31–1.01)	0.46 (0.14–1.51)	0.34 (0.14–0.84)^*^	1.01 (0.72–1.44)
Model I	Ref	0.55 (0.31–0.99)	0.47 (0.15–1.52)	0.33 (0.13–0.82)^*^	1.03 (0.72–1.45)
Model II	Ref	0.55 (0.31–0.99)	0.47 (0.14–1.52)	0.33 (0.13–0.82)^*^	1.10 (0.73–1.65)
Model III	Ref	0.65 (0.38–1.01)	0.47 (0.15–1.53)	0.34 (0.13–0.84)^*^	1.11 (0.74–1.66)
Mid-childhood, *n*	1998	275	75	171	613
Underweight					
*n* (%)	106 (5.3)	18 (6.5)	7 (9.3)	12 (7.0)	45 (7.3)
Crude	Ref	1.27 (0.74–2.17)	1.78 (0.75–4.25)	1.32 (0.69–2.54)	**1**.45 (0.98–2.15)
Model I	Ref	1.29 (0.76–2.21)	1.67 (0.69–4.06)	1.36 (0.70–2.62)	1.44 (0.96–2.12)
Model II	Ref	1.33 (0.77–2.28)	1.63 (0.68–3.97)	1.40 (0.74–2.69)	0.99 (0.60–1.62)
Model III	Ref	1.34 (0.79–2.33)	1.64 (0.66–3.97)	1.43 (0.74–2.79)	1.00 (0.61–1.63)
Overweight					
*n* (%)	211 (10.6)	26 (9.5)	9 (12.0)	16 (9.4)	62 (10.1)
Crude	Ref	0.77 (0.49–1.19)	0.61 (0.24–1.54)	0.60 (0.33–1.10)	1.01 (0.72–1.43)
Model I	Ref	0.76 (0.50–1.18)	0.62 (0.28–1.59)	0.59 (0.32–1.09)	0.97 (0.72–1.32)
Model II	Ref	0.77 (0.50–1.19)	0.63 (0.26–1.58)	0.59 (0.33–1.10)	1.00 (0.73–1.33)
Model III	Ref	0.77 (0.50–1.22)	0.63 (0.25–1.61)	0.60 (0.33–1.11)	1.02 (0.72–1.44)
Obese					
*n* (%)	145 (7.3)	15 (5.5)	4 (5.3)	6 (3.5)	50 (8.2)
Crude	Ref	0.78 (0.45–1.40)	0.89 (0.32–2.51)	0.55 (0.24–1.28)	1.05 (0.73–1.53)
Model I	Ref	0.77 (0.46–1.39)	0.94 (0.30–2.66)	0.54 (0.23–1.27)	1.09 (0.74–1.59)
Model II	Ref	0.76 (0.44–1.37)	0.96 (0.33–2.70)	0.54 (0.23–1.26)	1.20 (0.78–1.86)
Model III	Ref	0.80 (0.45–1.44)	0.98 (0.35–2.78)	0.59 (0.24–1.36)	1.23 (0.78–1.89)
Puberty, *n*	2059	286	75	177	641
Underweight					
*n* (%)	201 (9.8)	24 (8.4)	4 (5.3)	18 (10.2)	72 (11.2)
Crude	Ref	0.69 (0.42–1.14)	0.47 (0.15–1.51)	0.85 (0.47–1.53)	1.19 (0.87–1.61)
Model I	Ref	0.70 (0.40–1.16)	0.46 (0.15–1.46)	0.87 (0.46–1.52)	1.01 (0.63–1.46)
Model II	Ref	0.70 (0.42–1.18)	0.44 (0.14–1.44)	0.86 (0.47–1.57)	0.85 (0.55–1.30)
Model III	Ref	0.70 (0.43–1.18)	0.44 (0.13–1.45)	0.88 (0.48–1.59)	0.85 (0.57–1.24)
Overweight					
*n* (%)	208 (10.1)	31 (10.8)	4 (5.3)	16 (9.0)	68 (10.6)
Crude	Ref	1.17 (0.82–1.69)	0.84 (0.37–1.86)	1.08 (0.67–1.73)	1.14 (0.87–1.49)
Model I	Ref	1.15 (0.80–1.67)	0.85 (0.36–1.87)	1.07 (0.68–1.73)	1.16 (0.88–1.51)
Model II	Ref	1.16 (0.81–1.66)	0.84 (0.37–1.88)	1.07 (0.67–1.71)	1.30 (0.92–1.75)
Model III	Ref	1.21 (0.84–1.75)	0.84 (0.38–1.88)	1.13 (0.69–1.72)	1.32 (0.96–1.80)
Obese					
*n* (%)	142 (6.9)	17 (5.9)	6 (8.0)	12 (6.8)	58 (9.0)
Crude	Ref	0.88 (0.50–1.53)	1.01 (0.36–2.85)	0.99 (0.51–1.94)	1.34 (0.95–1.90)
Model I	Ref	0.90 (0.51–1.50)	1.03 (0.35–2.88)	0.99 (0.49–1.96)	1.33 (0.94–1.91)
Model II	Ref	0.86 (0.50–1.51)	1.03 (0.36–2.88)	0.98 (0.50–1.92)	1.34 (0.91–2.03)
Model III	Ref	0.95 (0.55–1.60)	1.05 (0.37–2.96)	1.08 (0.54–2.13)	1.39 (0.92–2.12)

^a^No infertility problems is defined as men without childlessness, infertility assessment and treatment;

^*^Statistical significance after Bonferroni correction

ref: reference.

Model I: adjustment for birth weight, birth weight for gestational age and mother’s smoking at the end of pregnancy;

Model II: Model I + adjustment for marital status until age 46;

Model III: Model II + adjustment for education and smoking at age 31

### Mid-childhood

Among boys in mid-childhood, 4.5% of those who were underweight and 31.7% of those who were obese remained underweight or obese in adulthood, respectively. Weight class in mid-childhood had no significant association with infertility assessment, male factor infertility, infertility treatment, childlessness (Table 3) or number of children (Fig. 3).

### Puberty

Among boys in puberty, 3.5% of those who were underweight and 41.1% of those who were obese remained underweight or obese in adulthood, respectively. Weight class in puberty did not associate with infertility assessment, male factor infertility or infertility treatment, childlessness (Table 3) or number of children (Fig. 3). There was an inverse J-shaped (i.e. quadratic) association between BMI at age 14 years and number of children in adulthood, i.e. boys with low and high BMI had fewer children than normal weight boys. This association vanished after adjusting for marital status (Fig. 2B).

## Discussion

In this population-based cohort study, our main observation was that boys who had a lower BW, who were born SGA and those who were underweight during early childhood had an increased risk of involuntary childlessness as adults. Furthermore, these boys had fewer children in adulthood. Especially association between BW/SGA and childlessness was highly influenced by the decreased probability to have a partner. In contrast, being overweight or obese during early childhood was associated with a decreased risk of infertility assessment, male factor infertility and infertility treatments in adulthood. According to our results, BMI after mid-childhood was not associated with infertility.

Our study involved a large, population-based cohort and incorporated data from birth until age 50 , as such, provided a unique opportunity to investigate associations between childhood growth and several fertility parameters in adulthood. The study population was ethnically homogeneous. The data on births were reliable as they were derived from the Finnish Medical Birth Register and the Population Register Centre, which together account for all births in Finland. All measurements from birth until adolescence – except BMI at age 14, which was used for curve estimation – were performed by trained professionals at every stage. Additionally, men who reported never having attempted to have children were excluded. Another novel contribution of the present work was that, in addition to analyzing specific childhood and adolescent age groups, childhood growth trajectory data and important physiological landmarks (AP and AR) were also assessed.

Our finding that lower BW and SGA, but not early GA, were associated with childlessness and a lower number of children are in line with a previous study showing that men with unfavorable fetal growth patterns *in utero*, especially men born SGA, were more likely to have decreased sperm counts (15, 16, 17). Also, higher BW for GA has been shown to associate with better sperm concentration in young adults (18). On the other hand, many contrasting results have been published (29, 34, 35). In our study, the association between lower BW and SGA with childlessness was lost after adjusting for marital status, which may indicate that reproductive performance of boys with lower BW and SGA is rather influenced by social factors, decreasing their opportunity to establish a relationship and therefore to have children. According to the literature and our study, the correlation between BW and overall fertility appears as a complex phenomenon wherein social factors should be considered (33, 36, 37, 38).

In early childhood, underweight boys had a higher risk of childlessness, whereas boys with overweight and obesity had a lower risk for infertility assessment, male factor infertility and treatment as adults, when compared to normal weight boys. BMI from mid-childhood onwards was not associated with indices of infertility. An Australian cohort study investigating the influences of growth and adiposity in childhood through adolescence on testicular function in adulthood showed that optimal BMI trajectory through childhood and adolescence was associated with a larger testicular volume and higher serum inhibin B and T in adulthood. They also showed that poor childhood growth was associated with an increased risk of smaller testes (17). Another study from the United States could not confirm these findings but showed that overweight and obesity in young men at age 20 years were associated with poorer sperm quality (18).

The mechanisms behind the association between BMI and fertility parameters in early childhood may be multifactorial. Soon after birth, levels of luteinizing hormone and follicle-stimulating hormone and consequently levels of testosterone increase in boys, peaking between 1 week and 3 months, decreasing thereafter gradually until 6–9 months of age (39). The testosterone peak is associated with penile and testicular growth and with the proliferation of gonadal cells. This so-called ‘mini-puberty’, first described in the 1970s (40), is an important developmental stage (41) that may have impact on reproductive capacity later in life (42, 43, 44), as there is a linear correlation between testosterone levels in mini-puberty with growth velocity in infancy and early childhood. In boys, lower levels of testosterone in the first 5 months of life were associated with lower fat-free mass/lean body mass ratio and higher adiposity during the subsequent 6 years (45). Moreover, in boys with Klinefelter syndrome, testosterone substitution in the early months increased total body mass, growth velocity and fat-free mass and reduced fat mass (46).

Mini-puberty and early childhood are complex developmental stages, marked by nutritional, genetic and epigenetic factors (39, 47). In our population, we were unable to determine adiposity and fat-free mass in infancy or childhood, being aware that BMI alone does not depict the whole-body composition. BMI in childhood is influenced by chronic illnesses and psychosocial environment that can strongly affect the growth (48, 49) and exert a negative impact on later fecundability (50, 51). Childhood growth restriction increases the likelihood of insulin resistance, metabolic syndrome and cardiovascular diseases in adulthood (47, 52, 53), which again are associated with infertility and lower levels of testosterone (10, 54, 55).

The present findings are in contrast to our results reported in girls from the same cohort (22) and in other female study populations (13, 14, 30, 56) in which mid-childhood and adolescence overweight and obesity have been associated with childlessness and decreased fecundability in adulthood. In girls, underweight in adolescence – but not at younger ages – has been associated with an increased risk of infertility treatment but not with childlessness in adulthood (22). This pattern did not occur among adolescent boys in the present study. Indeed, we found an inverse J-shaped correlation between number of children and BMI at age 14 years, which is in line with previous studies (13, 14). In our study, the significance was lost after marital status was considered, indicating again that social factors are strongly associated with growth patterns, infertility problems and family size from adolescence onwards.

In this study, AR at a younger age, but not BMI, was associated with childlessness, in line with our results obtained in girls in this same study population (22). Early AR has previously been linked to adverse metabolic outcomes and obesity (20, 21) and polycystic ovary syndrome in adult women (57). Based on our study, it seems that earlier age of AR in boys is associated with childlessness independently of marital status, but that BMI at the age of AR does not associate with infertility or childlessness later in life.

Our study has some limitations. Despite high participation rates at all data collection points, complete growth data were not available for all participants (missing *n* = 1149, i.e. 27% of the study population) and the lack of longitudinal data from AR onwards is a limitation of this study. As underweight and obesity were defined by the presence of at least one BMI value under or over the 95th pc in any age group, respectively, there was likely to be an over-diagnosis, especially in early childhood. Also, BMI does not remain constant throughout life and is especially prone to fluctuations from birth until the age at which AR occurs (58). Evidence from the current study supports the literature in this regard, as only 23% of boys in early childhood who had been classified as obese ‘at least once’ remained obese in adulthood; likewise, only 5.4% of those who had been considered underweight at least once remained underweight in adulthood. Another limitation of the present research was that data collection ended at age 50, even though male fecundity does not. However, according to the official statistics of our country, only 1% of men have their first child after 50 years of age (59). Infertility outcomes were self-reported, which can be considered as a limitation. Nevertheless, previous studies have shown a reliable correlation between self-reported fertility treatments and medical records (60, 61). Moreover, the self-reported data in the present study (male factor infertility rate: 28.5%; infertility treatment rate: 10.1%) are in line with the data from previous studies in other countries, which have indicated a 5–15% prevalence of infertility in the general population and a 20–30% rate for male infertility (7, 8). Last, childlessness, especially number of children, is certainly not fully valid measure for relative fertility, as social factors play a role in determining family size.

In conclusion, the current study revealed that boys with lower BW, SGA and lower BMI during early childhood were more likely to experience infertility-related problems and were at an increased risk of childlessness and having fewer children as adults. Unlike girls, boys with overweight or obesity in later childhood and puberty displayed no increased risk for infertility problems, childlessness or decreased number of children, after considering marital status. In boys, optimal growth during pregnancy as well as during early childhood seems to be very important for life-long fertility.

## Supplementary Material

Supplementary Figure 1: Directed acyclic graph in NFBC66: Fertility is outcome and primary exposure is childhood growth. This model has been used as a basis for logistic regression analyses.

Supplementary Table 1: Questions on fertility, marital status, education and smoking. Men who reported never having attempted to have children were excluded.

Supplementary Table 2: Association between infancy “underweight” (BMI under 5th pc) and “obesity” BMI over 95th pc) at ages 6 and 12 months and fertility outcomes compared to “normal weight” group (5th- 95th pc). Men who reported never having attempted to achieve pregnancy are excluded from the analyses. No infertility problems” was used as a reference group.

## Declaration of interest

Terhi T Piltonen is on the editorial board of the *European Journal of Endocrinology*. Terhi T Piltonen was not involved in the review or editorial process for this paper, on which she is listed as an author and in the decision to submit it for publication. The other authors have no conflicts of interest to disclose.

## Funding

NFBC1966 received financial support from University of Oulu Grant no. 65354, Oulu University Hospital Grant no. 2/97, 8/97, Ministry of Health http://dx.doi.org/10.13039/100009647 and Social Affairs Grant no. 23/251/97, 160/97, 190/97, National Institute for Health and Welfare, Helsinki Grant no. 54121, Regional Institute of Occupational Health, Oulu, Finland Grant no. 50621, 54231. Researches own funding: The Finnish Medical Foundation http://dx.doi.org/10.13039/100001236, the North Ostrobothnia Regional Fund, the Academy of Finland http://dx.doi.org/10.13039/501100002341 (project grants 315921, 104781, 120315, 129269,1114194, 24300796, 321763, 268336), Center of Excellence in Complex Disease Genetics and SALVE, the Sigrid Juselius Foundation, Biocenter Oulu, University Hospital Oulu and University of Oulu (75617), Jalmari ja Rauha Ahokkaan säätiö¨, The Finnish Medical Foundation http://dx.doi.org/10.13039/100001236, Medical Research Center Oulu http://dx.doi.org/10.13039/100016332, National Institute for Health Research http://dx.doi.org/10.13039/100005622 (UK), The European Union’s Horizon 2020 research and innovation program (under Grant agreement no. 633595 for the DynaHEALTH action and GA 733206 for LifeCycle). The funders had no role in study design, in the collection, analysis and interpretation of the data, in the writing of the article.
