# Supplementary figures and images for: An optimal growth pattern during pregnancy and early childhood associates with better fertility in men
Source: Eur J Endocrinol. 2022 Oct 13;187(6):847–58. doi: 10.1530/EJE-22-0385 (PMC9716397; doi:10.1530/EJE-22-0385)

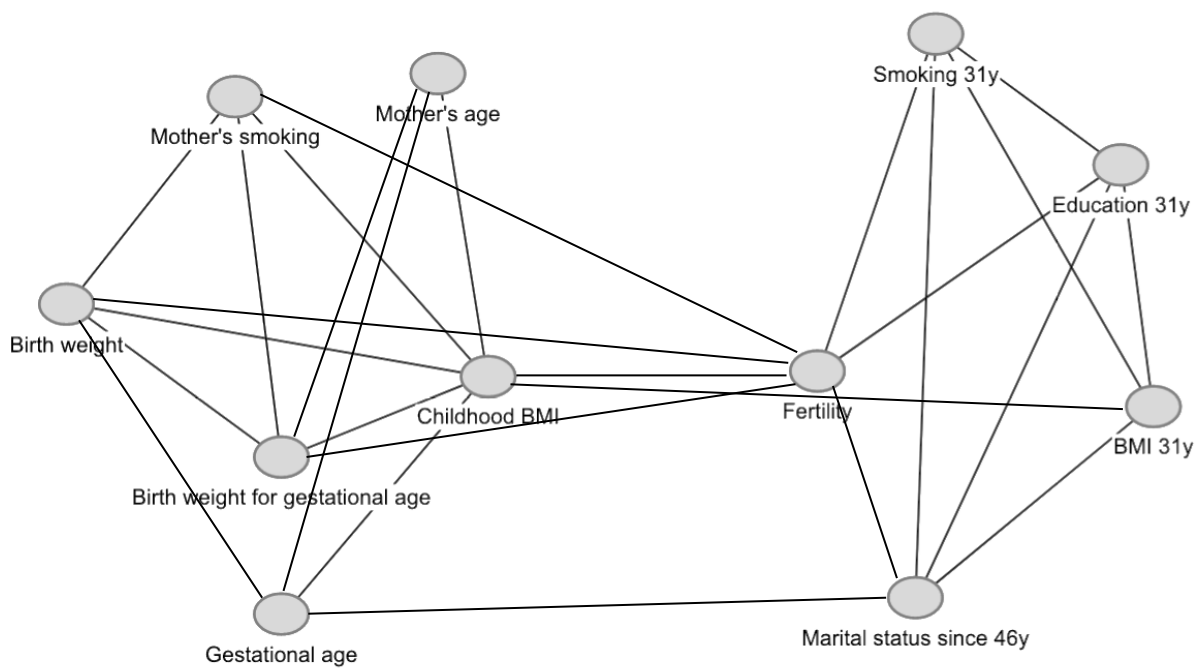

Supplement: Supplementary Figure 1: Directed acyclic graph in NFBC66: Fertility is outcome and primary exposure is childhood growth. This model has been used as a basis for logistic regression analyses. [file supplementary_figure_1.pdf]
