# Supplementary material for: An optimal growth pattern during pregnancy and early childhood associates with better fertility in men
Source: Eur J Endocrinol. 2022 Oct 13;187(6):847–58. doi: 10.1530/EJE-22-0385 (PMC9716397; doi:10.1530/EJE-22-0385)
Supplement: Supplementary Table 1: Questions on fertility, marital status, education and smoking. Men who reported never having attempted to have children were excluded. [file supplementary_table_1.pdf]

Supplementary Table 1: Questions on fertility, marital status, education and smoking. Men who reported never having attempted to have children were excluded.

	Questions	Parameters	Study population N=4128		
**Age 31**	Your educational status? Mark the highest.	**Education**<br>1)Basic education<br>2)Secondary education<br>3)Tertiary education	 N=468<br>N=2708<br>N=595 Missing N=357		
	Have you ever smoked in your life? Have you ever smoked regularly? Do you smoke nowadays?	**Smoking**<br>1)Never smoked<br>2)Former / occasional smoker<br>3)Active smoker	 N=1395<br>N=1016<br>N=1334 Missing N=383		
**Ages 31 and 46**			At age 31y	At age 46y	**Before 46y**
	Has infertility ever been a problem for you?	**1) No infertility problem**<br>**2) Infertility problem**<br>3) Never attempted to have children	N=3063<br>N=309<br>N=356<br>Missing N=400	N=2524<br>N=357<br>N=0<br>Missing N=1247	N=3654<br>N=474
	Have you been assessed for infertility?	**1) No infertility assessment**<br>**2) Infertility assessment**<br>3) Infertility assessment only for a partner (not included)	N=3374<br>N=207<br>N=87<br><br>Missing N=461	N=2424<br>N=305<br>N=68<br><br>Missing N=1331	N=3598<br>N=390<br>N=140
	Has the reason for your infertility been found?	**1) Yes, me or my partner and me = male factor infertility**<br>2) Yes, from my partner<br>3) No reason found	N=60<br><br>N=94<br>N=140	N=88<br><br>N=71<br>N=214	N=111<br><br>N=177<br>N=243
	Have you been treated for infertility?	**1) No infertility treatment**<br>**2) Infertility treatment**	N=3317<br>N=57<br>Missing N=754	N=2451<br>N=230<br>Missing N=1447	N=3869<br>N=259
	Your marital status: married, cohabiting, single, divorced, widowing with women?	**Marital status**<br>1)Ever been in relationship<br>2)Single	 N=2842<br>N=918<br>Missing N=138	 N=2605<br>N=333<br>Missing N=1190	 N=3462<br>N=666
