# Supplementary material for: An optimal growth pattern during pregnancy and early childhood associates with better fertility in men
Source: Eur J Endocrinol. 2022 Oct 13;187(6):847–58. doi: 10.1530/EJE-22-0385 (PMC9716397; doi:10.1530/EJE-22-0385)
Supplement: Supplementary Table 2: Association between infancy “underweight” (BMI under 5th pc) and “obesity” BMI over 95th pc) at ages 6 and 12 months and fertility outcomes compared to “normal weight” group (5th- 95th pc). Men who reported never having attempted to achieve pregnancy are excluded from the anal [file supplementary_table_2.pdf]

Supplementary Table 2: Association between infancy “underweight” (BMI under 5th pc) and “obesity” BMI over 95th pc) at ages 6 and 12 months and fertility outcomes compared to “normal weight” group (5th- 95th pc). Men who reported never having attempted to achieve pregnancy are excluded from the analyses. No infertility problems” was used as a reference group.

		**No infertility problems**[a]	**Infertility assessments before age 46**	**Male factor infertility before age 46 years**	**Infertility treatments before age 46**	**Childlessness at age 50**
Age 6 months	Total number	1908	261	78	180	613
BMI<5th pc	N (%)	126 (6.6)	18 (6.9)	7 (9.0)	15 (8.3)	50 (8.2)
	OR (Cl95%) Crude	ref	0.91 (0.51-1.62)	1.22 (0.48-3.10)	1.16 (0.62-2.16)	1.31 (0.92-1.89)
	Model I	ref	0.90 (0.50-1.62)	1.19 (0.45-3.06)	1.16 (0.60-2.15)	1.30 (0.91-1.89)
	Model II	ref	0.90 (0.51-1.61)	1.20 (0.46-3.08)	1.14 (0.61-2.14)	1.25 (0.86-1.87)
	Model III	ref	0.89 (0.50-1.57)	1.20 (0.46-3.06)	1.12 (0.61-2.09)	1.23 (0.81-1.92)
BMI>95th pc	N (%)	204 (10.7)	22 (8.4)	8 (10.3)	15 (8.3)	54 (8.8)
	OR (Cl95%) Crude	ref	0.76 (0.46-1.25)	1.04 (0.46-2.32)	0.72 (0.39-1.32)	0.80 (0.56-1.12)
	Model I	ref	0.76 (0.47-1.27)	1.07 (0.47-2.41)	0.72 (0.38-1.33)	0.80 (0.54-1.12)
	Model II	ref	0.77 (0.47-1.26)	1.06 (0.45-2.40)	0.72 (0.39-1.34)	0.75 (0.50-1.11)
	Model III	ref	0.80 (0.48-1.31)	1.02 (0.49-2.47)	0.74 (0.41-1.37)	0.75 (0.50-1.12)
Age 12 months	Total number	2438	333	95	222	809
BMI<5th pc	N (%)	23 (0.9)	5 (1.5)	1 (1.1)	3 (1.4)	10 (1.2)
	OR (Cl95%) Crude	ref	1.51 (0.51-4.46)	1.49 (0.19-11.37)	1.67 (0.49-5.68)	1.48 (0.69-3.18)
	Model I	ref	1.50 (0.52-4.45)	1.47 (0.18-11.35)	1.69 (0.49-5.73)	1.47 (0.68-3.10)
	Model II	ref	1.51 (0.52-4.46)	1.47 (0.19-11.28)	1.70 (0.51-5.80)	1.12 (0.45-2.85)
	Model III	ref	1.52 (0.51-4.55)	1.52 (0.20-11.75)	1.71 (0.50-5.89)	1.15 (0.45-2.86)
BMI>95th pc	N (%)	494 (20.3)	71 (21.3)	21 (22.1)	44 (19.8)	154 (19.0)
	OR (Cl95%) Crude	ref	1.00 (0.74-1.36)	1.00 (0.57-1.75)	0.92 (0.63-1.34)	0.89 (0.72-1.10)
	Model I	ref	1.00 (0.74-1.37)	1.02 (0.58-1.80)	0.93 (0.62-1.36)	0.88 (0.71-1.11)
	Model II	ref	1.00 (0.74-1.36)	1.02 (0.57-1.79)	0.93 (0.64-1.36)	0.86 (0.66-1.12)
	Model III	ref	1.02 (0.75-1.38)	1.04 (0.59-1.84)	0.93 (0.65-1.37)	0.87 (0.66-1.13)

[a] No infertility problems is defined as men without childlessness, infertility assessment and treatment.

BMI: body mass index, pc: percentile. Results are shown as Odds Ratios (OR) with 95% confidence intervals (Cl).

Model I: adjustment for birth weight, birth weight for gestational age and mother’s smoking at the end of pregnancy
Model II: Model I + adjustment for marital status until age 46
Model III: Model II + adjustment for education and smoking at age 31
